# Co‐occurrence of antibiotic, biocide, and heavy metal resistance genes in bacteria from metal and radionuclide contaminated soils at the Savannah River Site

**DOI:** 10.1111/1751-7915.13578

**Published:** 2020-05-03

**Authors:** Jesse C. Thomas, Adelumola Oladeinde, Troy J. Kieran, John W. Finger, Natalia J. Bayona‐Vásquez, John C. Cartee, James C. Beasley, John C. Seaman, J Vuan McArthur, Olin E. Rhodes, Travis C. Glenn

**Affiliations:** ^1^ Department of Environmental Health Science University of Georgia Athens GA 30602 USA; ^2^ Bacterial Epidemiology and Antimicrobial Resistance Research Unit United States Department of Agriculture Athens GA 30605 USA; ^3^ Department of Biological Sciences Auburn University Auburn AL 36849 USA; ^4^ Institute of Bioinformatics University of Georgia Athens GA 30602 USA; ^5^ Division of STD Prevention Centers for Disease Control and Prevention Atlanta GA 30329 USA; ^6^ Savannah River Ecology Laboratory University of Georgia PO Drawer E Aiken SC 29802 USA; ^7^ Warnell School of Forestry and Natural Resources University of Georgia Athens GA 30602 USA; ^8^ Odum School of Ecology University of Georgia Athens GA 30602 USA

## Abstract

Contaminants such as heavy metals may contribute to the dissemination of antimicrobial resistance (AMR) by enriching resistance gene determinants via co‐selection mechanisms. In the present study, a survey was performed on soils collected from four areas at the Savannah River Site (SRS), South Carolina, USA, with varying contaminant profiles: relatively pristine (Upper Three Runs), heavy metals (Ash Basins), radionuclides (Pond B) and heavy metal and radionuclides (Tim’s Branch). Using 16S rRNA gene amplicon sequencing, we explored the structure and diversity of soil bacterial communities. Sites with legacies of metal and/or radionuclide contamination displayed significantly lower bacterial diversity compared to the reference site. Metagenomic analysis indicated that multidrug and vancomycin antibiotic resistance genes (ARGs) and metal resistance genes (MRGs) including those associated with copper, arsenic, iron, nickel and zinc were prominent in all soils including the reference site. However, significant differences were found in the relative abundance and diversity of certain ARGs and MRGs in soils with metal/radionuclide contaminated soils compared to the reference site. Co‐occurrence patterns revealed significant ARG/MRG subtypes in predominant soil taxa including *Acidobacteriaceae*, *Bradyrhizobium*, *Mycobacterium*, *Streptomyces*, *Verrumicrobium*, *Actinomadura* and Solirubacterales. Overall, the study emphasizes the potential risk of human activities on the dissemination of AMR in the environment.

## Graphical abstract

We present a comprehensive analysis of the soil microbiome in several areas affected by legacies of heavy‐metal and radionuclide contamination. We explore how these contaminants affect bacterial community composition and diversity, and the enrichment of antibiotic, biocide, and metal‐resistance genes. We find that these contaminants can not only have a profound impact on soil microbiome composition, but it can also affect how it functions.

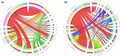

## Introduction

The prevalence and dissemination of antimicrobial resistance is a serious global public health concern that cannot be understated. Over the past 20 years, there has been a substantial increase in the incidence of clinical bacterial strains that have developed and acquired resistance to β‐lactams, quinolones, macrolides and lincosamides, and even to antibiotics of last resort such as carbapenems (Ventola, 2015; Klein *et al.*, 2018). According to the World Health Organization’s (WHO) 2014 report, the world is at the brink of a post‐antibiotic era, characterized by a significant reduction in the effectiveness of antibiotics and other antimicrobial agents, in addition to more frequent antibiotic treatment failures. Although there have been efforts worldwide aimed at reducing antibiotic usage, fostering antibiotic stewardship and monitoring the spread of antimicrobial resistance, these strategies have had limited success (Andersson and Hughes, 2010; Pruden *et al.*, 2013). Indeed, the current body of evidence suggests that the evolution and dissemination of antimicrobial resistance is shaped by a complex array of factors, many of which are independent of antibiotic usage (Baker‐Austin *et al.*, 2006; Pal *et al.*, 2015). Previous studies have demonstrated that heavy metal contamination in the natural environment can play an important role in the maintenance and proliferation of antibiotic resistance (Stepanauskas *et al.*, 2006; Seiler and Berendonk, 2012; Wales and Davies, 2015; Li *et al.*, 2017). This co‐selection phenomenon is the result of antibiotic and metal resistance phenotypes sharing many overlapping genetic mechanisms including: co‐resistance (close linkage between multiple different resistance genes), cross‐resistance (single genetic elements that regulate both antibiotic and metal resistance genes) and co‐regulation (shared regulatory system to antibiotic and metal resistance; Baker‐Austin *et al.*, 2006).

Anthropogenic activities (e.g., industry and agriculture) have greatly contributed to release of large quantities of heavy metals, impacting both soil and water (Wei and Yang, 2010; Wuana and Okieimen, 2011; Gonçalves *et al.*, 2014; Su *et al.*, 2014). This is particularly concerning given that unlike antibiotics, heavy metals do not degrade in the environment and can exert long‐standing and widespread co‐selection pressure (Baker‐Austin *et al.*, 2006). Moreover, several studies have reported on the co‐selection of antibiotic resistance genes (ARGs) and metal resistance genes (MRGs) in a variety of contaminated environments. These studies have also demonstrated how such genes can be enriched and disseminated in various animal models, sediment, bodies of water and even the human gut (Cesare *et al.*, 2016; Fang *et al.*, 2016; Henriques *et al.*, 2016; Ju *et al.*, 2016; Zhao *et al.*, 2018). Considering the overarching global health problem of antibiotic resistance, it is critical to understand the distribution and diversity of resistance genes, and their hosts, in the environment.

**Fig. 1:**
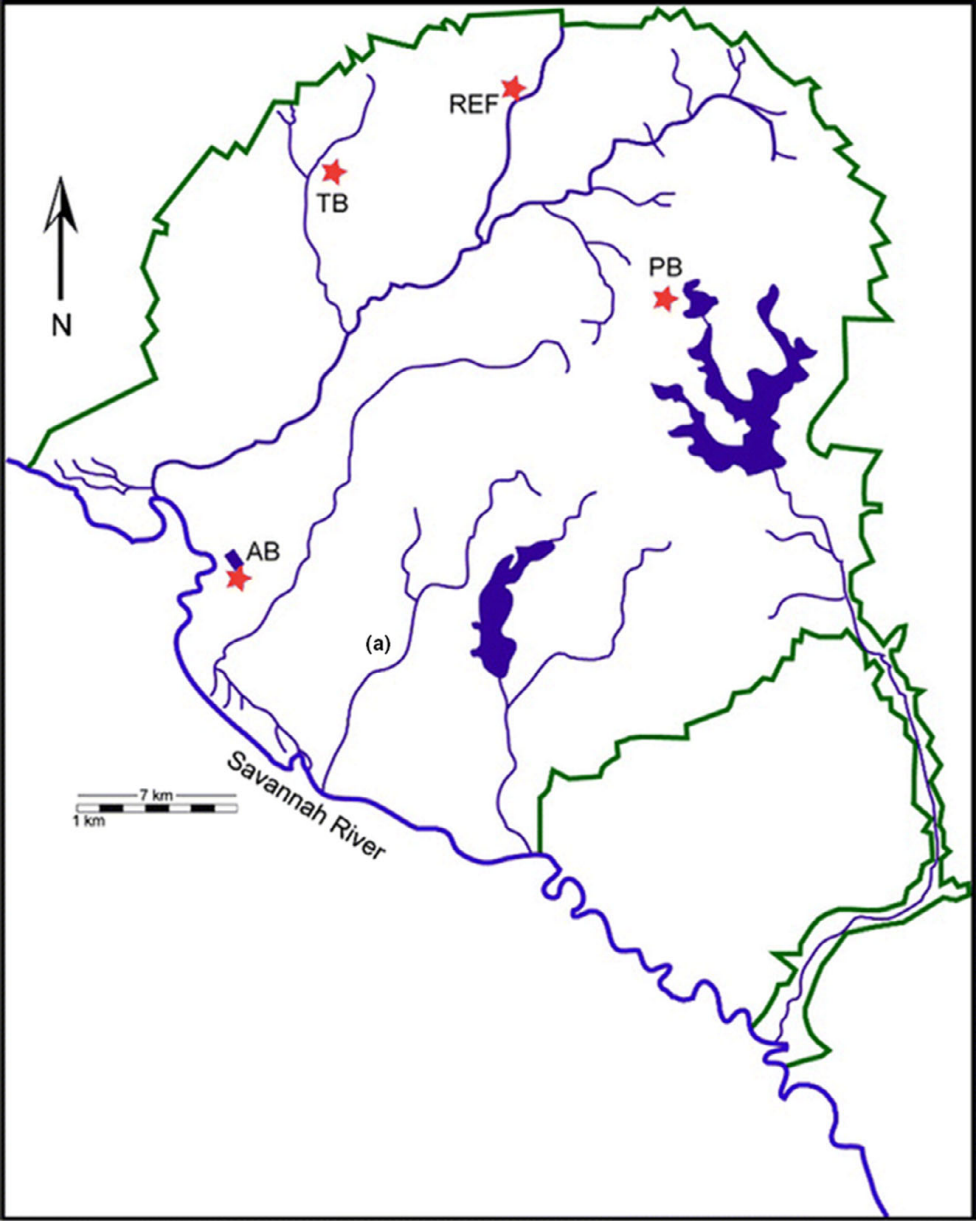
Map of the Savannah River Site, South Carolina, USA, depicting the distribution of the reference location (Upper Three Runs – UR) and contaminated study areas, ash basin (AB), Pond B (PB) and Tim’s Branch (TB) sampled for soil in spring 2014.

The Savannah River Site (SRS, 33°00′N and 81°41W) is an approximately 800 km^2^ former nuclear weapons production facility situated along the Savannah River in the upper coastal plain of South Carolina near Aiken, SC (Fig. 1). The SRS is characterized as having humid, subtropical weather (Garten *et al.*, 2000). Historically, the SRS served as the Department of Energy (DOE) production and refinement facility for nuclear materials. As part of its mission between the 1950 through 1980s the SRS operated five nuclear reactors, nine coal‐fuelled power stations, a heavy‐water extraction plant, nuclear fuel and target fabrication areas, waste management facilities, and additional research and administrative facilities located across the site landscape (Seaman *et al.*, 2007; Seiler and Berendonk, 2012; Baker *et al.*, 2019). Over the years, a combination of routine operations, improper disposal practices and incidental spills contributed to the release of organic and inorganic waste into this environment. Some of the most widespread contaminants included heavy metals, metalloids and radionuclides which were stored in massive storage tanks, discharged to unlined seepage basins or buried in shallow trenches (Seaman *et al.*, 2007). In 1989, the site was officially listed on EPA’s National Priorities List (NPL) due to chemical (solvents), heavy metal (HMs) and radionuclide contamination of on‐site groundwater (Agency for Toxic Substances and Disease Registry, 2007).

Previous studies on the relationship between heavy metal contamination and microbial communities at the SRS have mainly relied on the use of culture‐dependent methods such as microcosms, Biolog EcoPlates™ plates or Sensitre™ panels to examine co‐selection of antibiotic resistance (Stepanauskas *et al.*, 2006; McArthur *et al.*, 2016, 2017; Agarwal *et al.*, 2019). In the current study, we utilized microbiome and metagenomic analyses to examine soil habitats at the SRS. Specifically, we explored differences between soil microbial communities in non‐impacted and metal‐ and radionuclide‐impacted sites, examined the abundance and co‐occurrence of ARGs and MRGs, and unraveled the predominant bacterial hosts where co‐resistance is found.

## Results

### General characteristics of the soil samples

Soil samples displayed significant differences with respect to edaphic properties including water content, acidity, total P content and C:N ratio (Table 1). Soils from Upper Three Runs were the most acidic, showed the highest ratio of C:N, the second highest water content and the third most total P. The Pond B soils were the least acidic, exhibited the highest water content, and lowest total P, C and N. Soils from Tim’s Branch had a significantly lower pH compared to the soils from Pond B and Ash Basins (*P* < 0.05). Tim’s Branch soils also exhibited the lowest water content of all the soils, and second highest total P, C, N. The soils from Ash Basins exhibited the second highest pH, second lowest water content, highest total P and third highest C to N ratio.

**Table 1:** Soil heavy metal concentrations and edaphic properties.

Heavy metal (mg kg^−1^) or edaphic factor	Soil concentrations (mean ± SD)			
	Upper three runs (reference site) 33.37055N 81.62907W	Ash basins 33.19474N W81.73683 W	Tim's branch N33.32664 N W81.71932 W	Pond B N33.29394 N W81.54306 W
Chromium	17.42 ± 10.83	25.75 ± 22.36	19.28 ± 10.63	12.3 ± 6.42
Cobalt	2.74 ± 1.27	4.55 ± 5.1	5.71 ± 3.38	1.52 ± 0.39
Nickel	6.64 ± 4.43	9.75 ± 11.64	8.99 ± 6.41	5.32 ± 3.95
Cupper	7.49 ± 5.92	11.65 ± 16.18	7.23 ± 3.54	4.22 ± 2.6
Zinc	22 ± 14.37	25.07 ± 23.55	20.77 ± 10.89	11.64 ± 3.44
Arsenic	26.63 ± 2.48	27.33 ± 4.88	25.99 ± 1.59	24.8 ± 1.65
Strontium	14.46 ± 9.33	40.34 ± 49.25	25.05 ± 13.1	5.92 ± 2.95
Lead	21.48 ± 14.75	16.42 ± 11.03	18.59 ± 9.1	7.74 ± 4.88
Uranium	1.61 ± 0.88	2.11 ± 1.38	6.29 ± 13.77	1.03 ± 0.31
Phosphorus (mg g^−1^)	0.01 ± 0	0.02 ± 0	0.01 ± 0	0 ± 0
pH	3.98 ± 0.03	4.27 ± 0.04	4.34 ± 0.01	4.38 ± 0.03
Moisture (g)	22 ± 0	8.33 ± 0.58	7 ± 0	29.33 ± 0.58
Carbon (mg g^−1^)	12.67 ± 0.87	5.8 ± 0.36	11.38 ± 1.85	3.11 ± 0.43
Nitrogen (mg g^−1^)	0.48 ± 0.04	0.31 ± 0.02	0.58 ± 0.1	0.11 ± 0.02
Carbon to Nitrogen Ratio	26.78 ± 0.47	18.57 ± 0.01	19.68 ± 0.52	27.82 ± 0.15

### Metal concentrations in soils

Heavy metal concentrations in soils from the four sampling sites are provided in Table 1. Pairwise comparisons varied depending on the type of heavy metal, however, generally, metals such as Strontium [Ash Basins: up to 176.27 mg kg^−1^ (40.43 ± 49.24 mg kg^−1^), Tim’s Branch: up to 37.90 mg kg^−1^ (25.05 ± 13.10 mg kg^−1^)] and Cobalt [Ash Basins: up to 18.17 mg kg^−1^ (4.53 ± 5.10 mg kg^−1^), Tim’s Branch: up to 12.99 mg kg^−1^ (5.71 ± 3.38 mg kg^−1^)] were significantly higher (*P* < 0.05) in soil samples from Ash Basins and Tim’s Branch, compared to Pond B and Upper Three Runs (Table 1). It should be noted that although we did not detect significant differences in heavy metal concentrations between soils from Pond B and Upper Three Runs, the vast majority of heavy metal and radionuclide contamination at the Pond B reservoir are bond to the pond’s sediments and, thus, may explain the lower values compared to the reference site (Stephens *et al.*, 1998; Lord *et al.*, 2002).

### Soil archaeal and bacterial communities

**Fig. 2:**
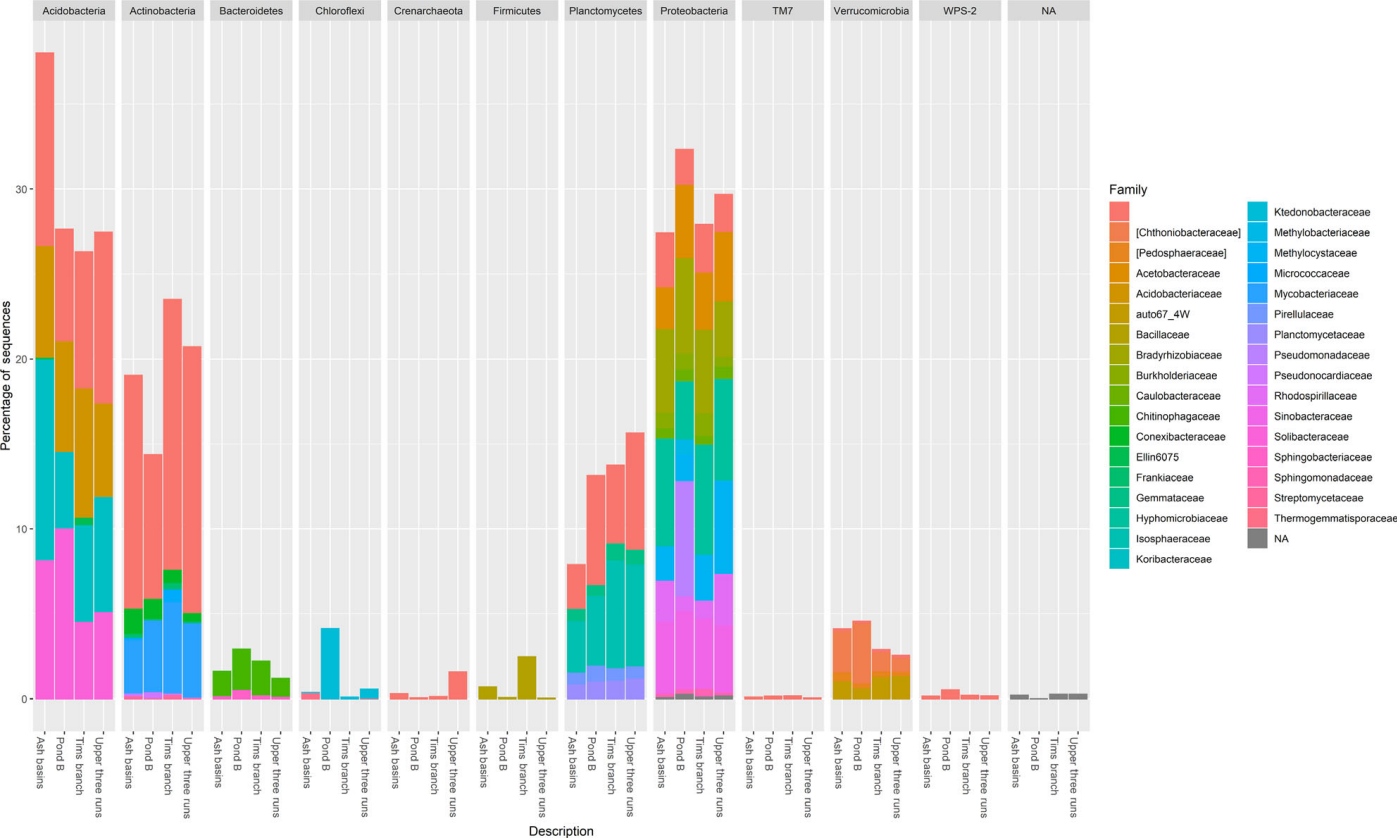
Relative abundance at the level of Phylum and corresponding families representing the nine most abundant bacterial/archaeal OTUs (clustered at 97% similarity) in soil samples from the four sampling sites. Bacterial phyla are further expanded into respective families.

After completion of quality filtering steps, denoising and chimera removal, a dataset of 6 078 406 high‐quality (*Q* ≥ 20) 16S rRNA gene sequences with an average read length of 458.2 ± 18.6 bp were obtained for the 80 samples for further analyses. Analyses were performed on rarefied data using an even sampling depth of 15,000 reads per sample. A total of 311 376 OTUs (16655.36 ± 8030.35 OTUs/sample) were identified spanning three archaeal phyla (15 orders, 17 families) and 51 bacterial phyla (574 orders and 559 families). For archaea, the two dominant phyla included Crenarchaeota (0.35% ± 0.40%) and Euryarchaeota (0.15% ± 0.14%). Within those phyla, we identified two dominant archaeal classes from the soils, Methanomicrobia (0.096% ± 0.108%) and Marine benthic group A (MGBA; 0.037% ± 0.048%). In addition, we also identified a core microbiome consisting of eight bacterial phyla (i.e., Acidobacteria, Actinobacteria, Bacteroidetes, Firmicutes, Planctomycetes, Proteobacteria, TM7, Verrucomicrobia, WPS‐2) shared among 95% of the sequences analysed in this study. In terms of relative abundance, the dominant phyla (sequences with an abundance > 1%) across the four soil sites were Proteobacteria (27.24% ± 0.02%), Acidobacteria (22.00% ± 0.04%), Actinobacteria (14.83% ± 0.02%), Planctomycetes (11.65% ± 0.01%), Verrucomicrobia (4.25% ± 0.01%), Bacteroidetes (3.00% ± 0.01%), Chloroflexi (1.85% ± 0.01%), TM7 (1.32% ± 0.00%) and Firmicutes (1.02% ± 0.01%; Fig. 2). Furthermore, deblurring revealed that the most abundant shared phylotypes that could be assigned to a genus/species were *Rhodoplanes* (Alphaproteobacteria; 3.20% ± 0.01%), *Candidatus solibacter* (2.84% ± 0.01%; Acidobacteria), *Mycobacterium celatum* (1.94% ± 0.00%), *Pseudomonas* (Gammaproteobacteria; 1.77% ± 3.52%) and *Candidatus xiphinematobacter* (1.38% ± 0.01%).

We detected significant differences in a myriad of soil taxa between sampling locations using a significance level of 0.05 (Kruskal–Wallis test using *group_significance.py*, Table S1). These included the taxonomic orders Desulfuromonadales (Proteobacteria), Lactobacillales (Firmicutes), Bacillales (Firmicutes), Coriobacteriales (Actinobacteria), Ktedonobacteria (Chloroflexi), Acidobacteriales (Proteobacteria) and Pseudomonadales (Proteobacteria) among many others (Table S1). We used a non‐parametric test to statistically compare differences in taxonomic abundance of dominant bacterial orders with respect to the reference site using a significance level of 0.05. Compared with samples from Upper Three Runs, soils from Ash Basins displayed significantly higher abundances of several bacterial orders including Acidobacteriales, Solibacterales (Acidobacteria), Chthoniobacterales (Verrucomicrobia), Solirubrobacterales (Bacteroidetes), Sphingobacteriales (Bacteroidetes), Bacillales among others (Table S2). Soils from Pond B had significantly higher abundances of Solibacterales (Acidobacteria), Coriobacteriales, Solirubacterales, FW68 (Armatiomonadetes), Sphingobacteriales, Lactobacillales (Table S3). Soils from Tim’s Branch had significantly higher abundances of Burkholderiales (Proteobacteria), Saprospirales (Bacteriodetes), Bacillales, Solirubacterales, unassigned TM7‐1 (TM7), Sphingobacteriales, iii1‐15 (Acidobacteria) and Legionellales (Proteobacteria; Table S4).

### Soil environments with elevated heavy metal contamination display significantly reduced bacterial species richness

**Fig. 3:**
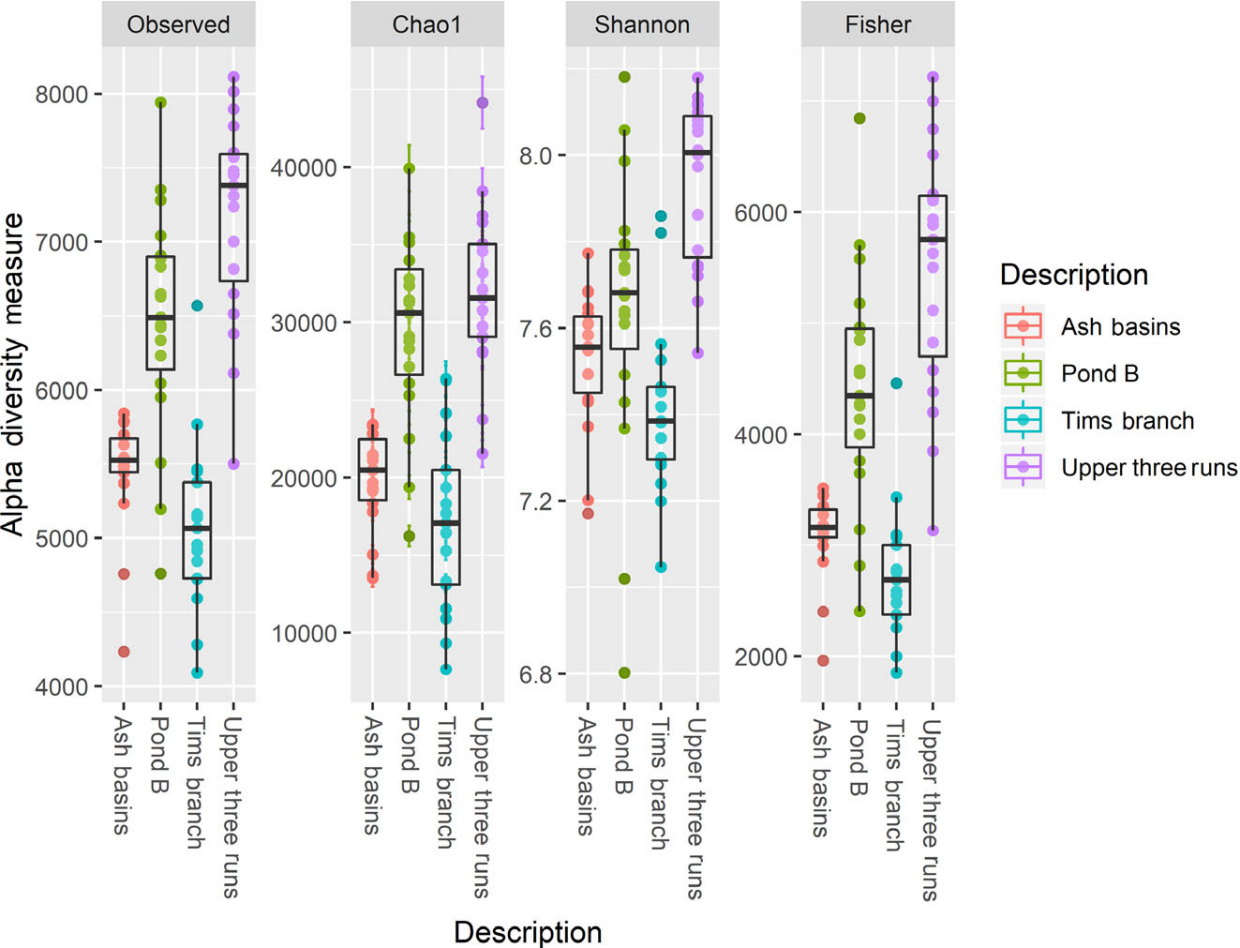
Alpha diversity measures in soil samples at the four sites (defined either by the number of bacterial/archaeal OTUs observed or by Chao1, ACE, Shannon, Inverse Simpson, and Fisher diversity measures).

Generally, soil samples from Tim’s Branch and Ash Basins displayed the lowest alpha diversity, while those from Pond B and Upper Three Runs had the highest diversity across all alpha diversity metrics (Fig. 3). In addition, when performing pairwise group comparisons there were significant differences in Chao1 and Faith’s alpha diversity indices when comparing the sites either Ash Basins or Tim’s Branch to Pond B and Upper Three Runs (non‐parametric test with *compare_alpha_diversity.py*, *P* < 0.05). There were no significant differences in Chao1 or Faith’s alpha diversity between Pond B compared to Upper Three Runs, or Tim’s Branch compared to Ash Basins.

### Drivers of bacterial community structure

**Fig. 4:**
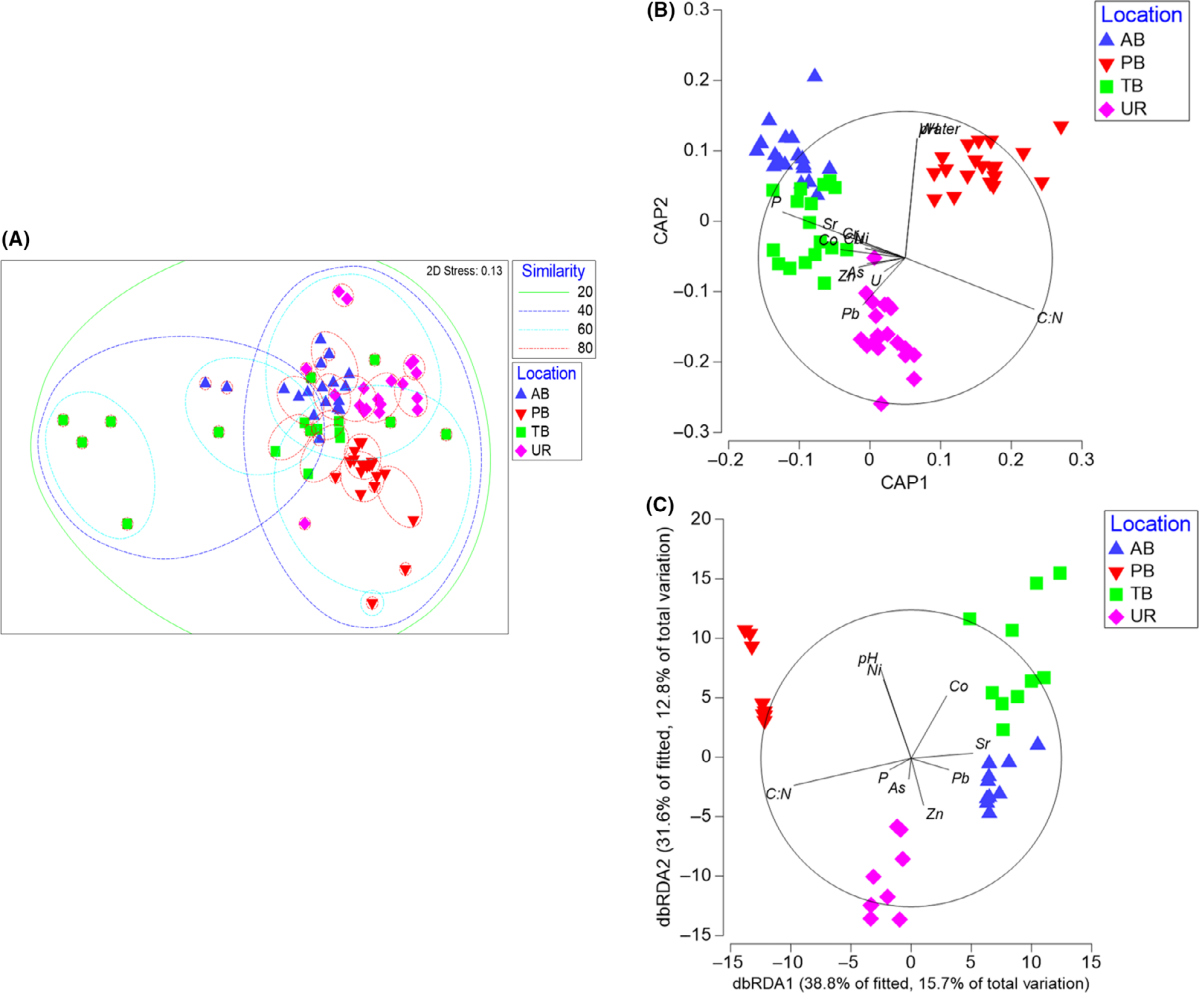
Structure of microbial soil communities at SRS. A. Non‐metric multi‐dimensional scaling plot of bacterial/archaeal OTU frequency after log‐transformation, which reduces the influence of the most abundant OTUs. Dashed lines represent per cent similarity of clusters using SIMPROF: green lines 20%, dashed blue lines 40%, dashed cyan lines 60%, dashed red lines 80%. B. Canonical analysis of principal coordinates based on a Bray–Curtis dissimilarity matrix of log‐transformed bacterial/archaeal OTU frequencies. C. Distance‐based redundancy analysis (dBRDA) representing raw Pearson correlations for habitat variables and bacterial/archaeal OTUs. Vectors are overlaid to represent the different HMs and edaphic factors most important to the modelling approach. Length and direction of vectors indicate the strength and direction of the relationship. Fitted variation refers to variance within the linear model created during the DistLM analysis. The total variation refers to the variance within the original data. Blue triangles represent soils from Ash Basins (AB); Red upside‐down triangles represent Pond B (PB) soils; Green squares represent Tim’s Branch (TB) soils; pink diamonds represent Upper Three Runs (UR) soils.

Multivariate analyses based on the Bray–Curtis distances between 16S rRNA gene profiles for the four sites revealed that the sampling region had a significant effect on the observed OTUs between samples (PERMANOVA, Pseudo‐*F* = 10.75, *P* = 0.001; Table S5). Unconstrained NMDS plots based on a Bray–Curtis dissimilarity matrix were overlaid with SIMPROF‐based cluster analysis data to examine overall similarity between sites in multi‐dimensional space (Fig. 4A). The NMDS plots displayed that overall the similarity between all soil groups was approximately 40% with many differences in within‐group variability. Because group differences can potentially be masked by high variability and high correlation structure among unrelated variables, we also applied CAP (Anderson and Willis, 2003). The results of the CAP ordination demonstrated that both the first squared canonical correlation (*σ*_1_^2^ = 0.9549) and second squared canonical correlation *(σ*_1_^2^ = 0.9301) were large, indicating that the soil bacterial OTUs correlated strongly with both canonical axes. Our CAP demonstrated bacterial communities clustered away from each other using location as the primary factor (our ‘*a priori hypothesis*’), with a slight overlap between samples from Tim’s Branch and Ash Basins (Fig. 4B). Vectors overlaid on the CAP plot indicated that P, Sr, Co, Ni, Cu, Zn and As, were more influential in shaping bacterial communities in Tim’s Branch and Ash Basins soils, while moisture content and pH were more influential in bacterial communities in Pond B soils. Lead was influential in shaping bacterial communities in Upper Three Runs soils.

We were specifically interested in fitting the edaphic properties (soil pH, moisture content, total P and C:N ratio) as well as the soil heavy metal concentrations to the dbRDA ordination. Our DistLM analysis revealed that pH, C:N ratio, Co, Ni, As, Zn, Sr and Pb explained 40% (*R* ^2^ sequential) of the observed total variation in the composition of the bacterial community in the soils (Table S6). Using these factors, the primary and secondary axes of the dbRDA explains 28.5% of the total variation, and 70.5% of the fitted variation (Fig. 4C). In general, the longer vectors for soil moisture content and C:N ratios suggest these variables are strongly correlated with the bacterial community structure at Pond B. The vectors for moisture content and Zn suggest these variables are strongly correlated with the community structure at Upper Three Runs. The soils from Ash Basins and Tim’s Branch were more strongly correlated with HMs, especially Sr, however, there were some exceptions. Cobalt was more influential in community structure in Tim’s Branch soils, while Zn and Pb were larger factors in Ash Basin soils.

### Inferring soil environmental metagenomes using PICRUSt

The mean weighted nearest sequenced taxon index (NSTI), which measures the prediction accuracy of PICRUSt, for our soil samples was 0.17 ± 0.02. We initially examined the relative abundance of predicted genes for metal and antibiotic resistance. Two KOs related to ARGs and MRGs, *emrB* (K07644) and *cusS* (K07644) respectively, were significantly enriched in soils from Ash Basins and Pond B compared to Upper Three Runs. Interestingly, the soil samples from Tim’s Branch contained the greatest number of predicted genes compared to Upper Three Runs. Predicted genes corresponding to Cu resistance (K14166; K06189) and antibiotic resistance (K07788, K07789), among several others, were significantly enriched in Tim’s Branch soils. A closer inspection of KOs revealed OTUs that contributed the largest proportion of predicted MRG and ARG‐like genes according to the 16S rDNA sequences included bacterial taxa such as Ktedonobacteraceae, Solibacteracea, *Pseudomonas viridiflava*, *Pseudomonas* spp*.*, Spingomonadales, Bradyrhizobiaceae and Spartobacteria (*metagenome_contributions.py*, Table S7).

### Metagenome sequencing and assembly summary

In total, we obtained 1.19 × 10^12^ high‐quality reads from the soil metagenomes, which were assembled into 1.17 × 10^9^ contigs with an average read length of 303 bp (Table S8). The assemblies contained 1.2 × 10^9^ open‐reading frames (ORFs) with an average length of 250 bp (Table S9). We identified 720 923 ARG‐like ORFs using the SARGfam v.2.0 database and 1 387 193 antimicrobial/biocide efflux and MRG‐like ORFs using the BacMet v.2.0 database (Table S9).

### Differences in ARG abundance between soils

In total, we detected an average of 0.43 ARG genes per 16S rRNA copy (Table S5). The predominant ARG types included multidrug resistance genes (52%), vancomycin resistance genes (28%) and fosmidomycin resistance genes (5%; Table S10). Overall, the metagenomic analyses indicated that ARG types were similar across soil habitats with no significant differences. The relative abundance of ARG types was lowest at Ash Basin’s ranging from 0.34 to 0.43 ARG genes per 16S rRNA copy; whereas they were higher at Pond B where they ranged between 0.37 and 0.57 ARG genes per 16S rRNA copy (Table S7). Significant differences were observed between the soil habitats for the following ARG types: aminoglycoside, β‐lactamases, chloramphenicol, fosfomycin, rifamycin and tetracycline resistance genes (*P* < 0.05; Table S11).

**Fig. 5:**
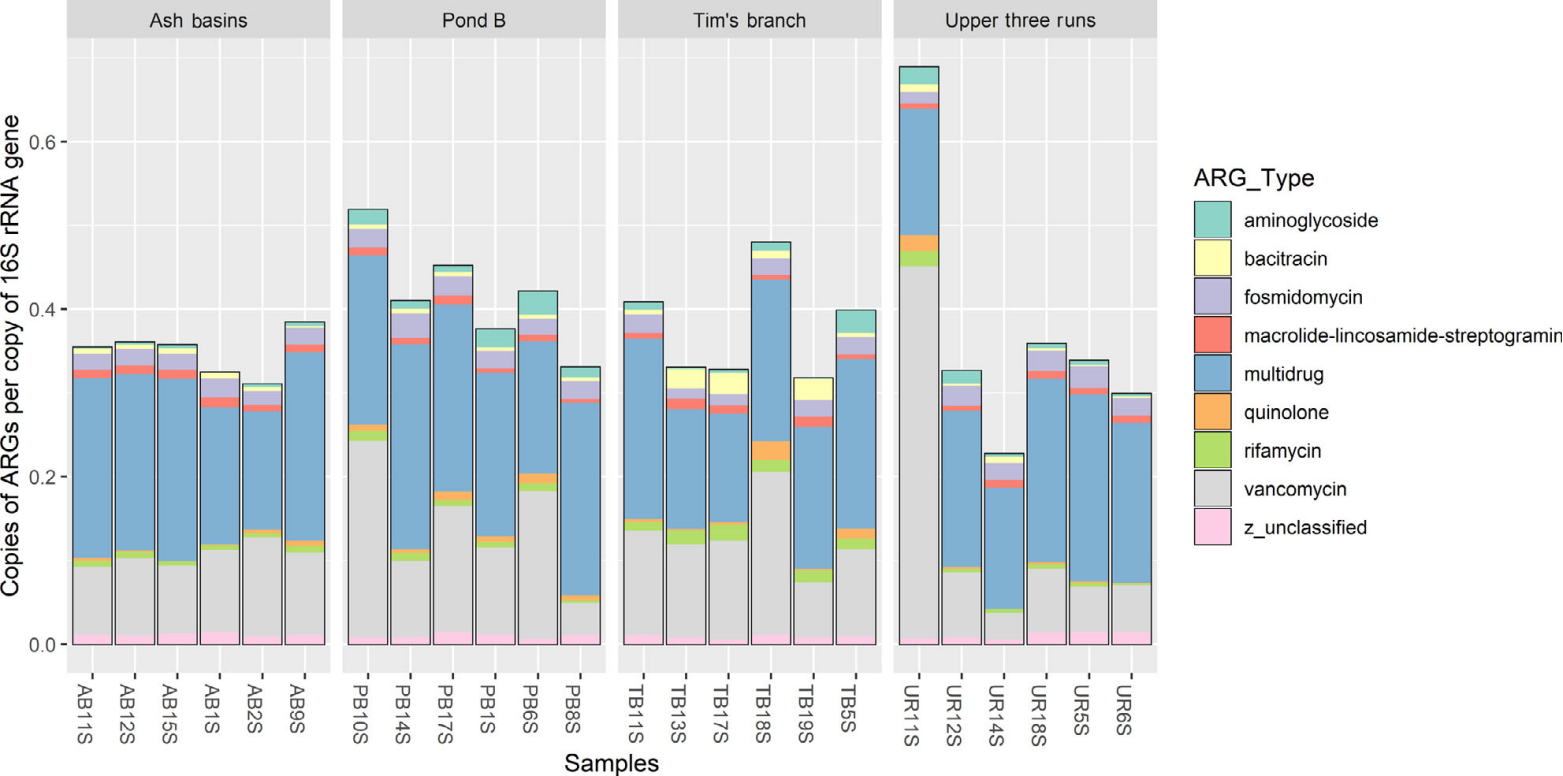
Bar plots showing the relative abundance of blast hits for the most abundant ARG‐types observed in all soil samples.

**Fig. 6:**
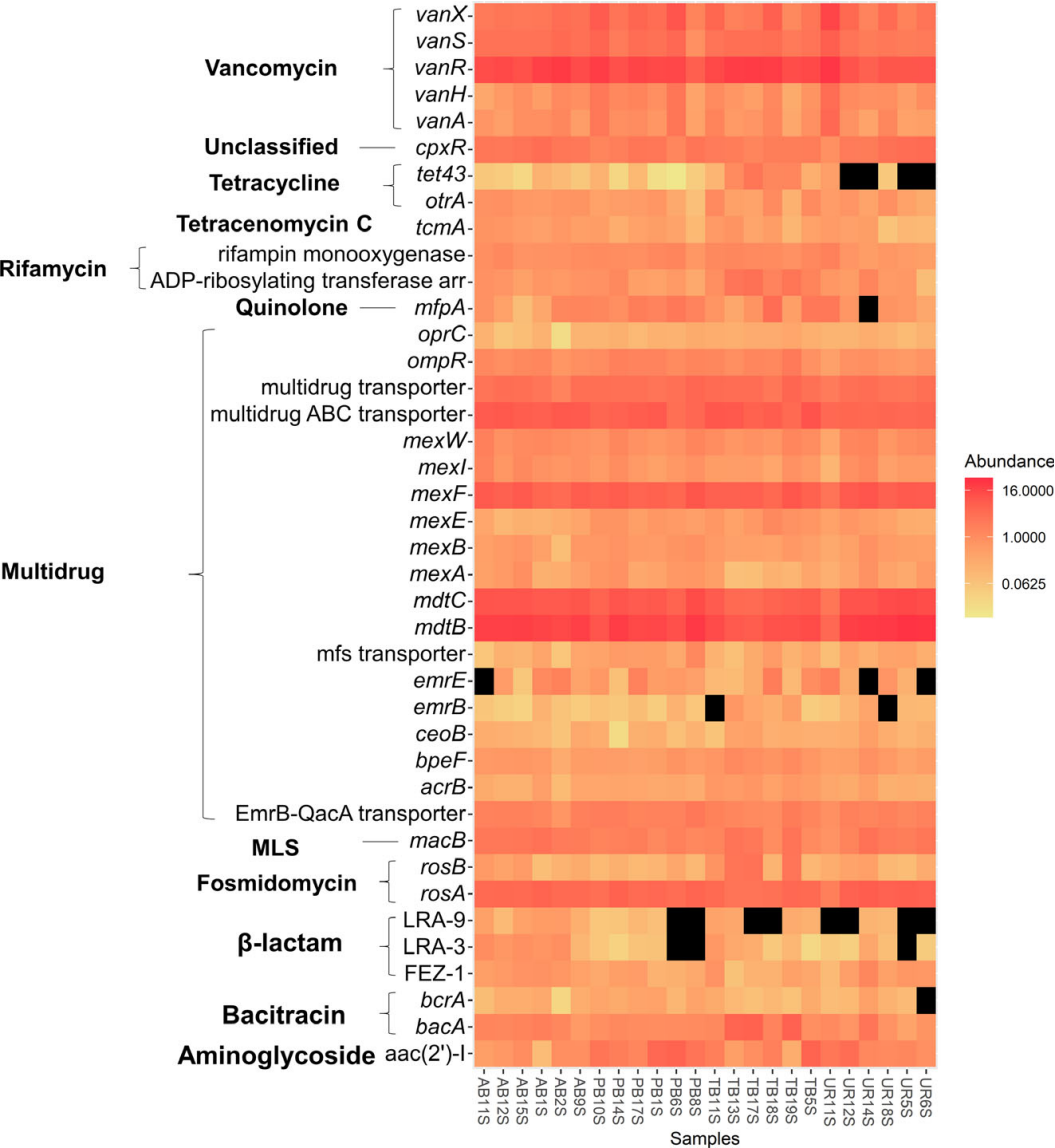
Heat map of variation of ARG‐like contigs contained in the top 40 ARG subtypes. Data is based on the relative abundance of blast hits (SARGfam) for each respective sample.

**Fig. 7:**
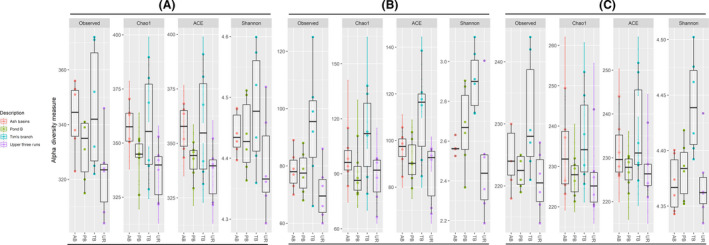
Alpha diversity measures from soils at the four sampling sites defined either by, (A) the number ARGs identified in SARGfam v.2.0, (B) antimicrobial/biocide efflux resistance genes identified in BacMet v.2.0 database, and (C) MRGs identified in BacMet v2.0 database using either the observed, Chao1, ACE or Shannon diversity measures.

The ARGs abundance data revealed that resistance genes primarily involved two‐component and multidrug transporters (Figs 5 and 6). The vancomycin resistance gene *vanR* and multidrug resistance gene *mdtB* were the two most abundant ARG subtypes. In fact, several genes from the VanS‐VanR two‐component regulatory system that encode the *van* gene cluster were identified in the top 20 ARG subtypes including *vanX*, *vanS* and *vanH* (Arthur *et al.*,  1992). Whereas, the multidrug resistance genes included several from the Resistance Nodulation Division (RND) family of transporters such as *mdtB*, *mdtC*, *mexF*, *emrE*, *omprR* and *acrB*. We identified several other ARG subtypes that appeared in the top 20. The fosmidomycin resistance gene *rosA*, identified in *Yersinia* spp., encodes a polypeptide similar to other genes involved in drug efflux. The macrolide‐lincosoamide‐streptogramin resistance gene, *macB*, is part a drug efflux system in the ATP‐binding cassette (ABC) family of transporters. The quinolone resistance gene *mfpA*, which impedes DNA‐binding to gyrase, the resistance gene *aah* (2′)‐I, which is involved in the covalent modification of aminoglycosides, and a plasmid‐mediated bacitracin resistance gene (*bacA*) were also detected. Shannon indices were used to examine the diversity of ARGs types between soils and our data indicated ranges between 4.29 and 4.60, with only soils from Tim’s Branch displaying significantly higher indexes compared to the reference site (*P* < 0.05; Fig. 7A; Table S12).

### Differences in antimicrobial/biocide efflux and MRG abundance between soils

**Fig. 8:**
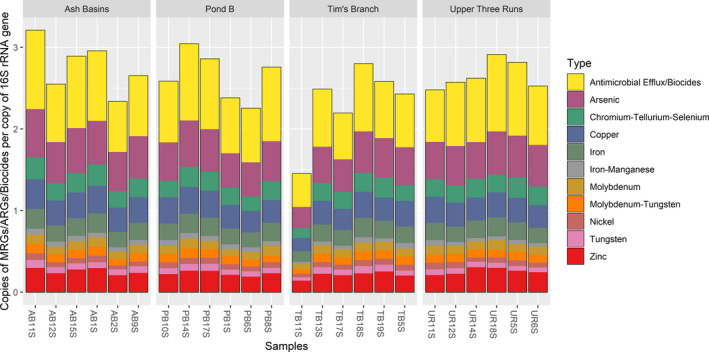
Bar plots showing the relative abundance of blast hits for the most abundant AB‐MRG types observed in all soil samples.

**Fig. 9:**
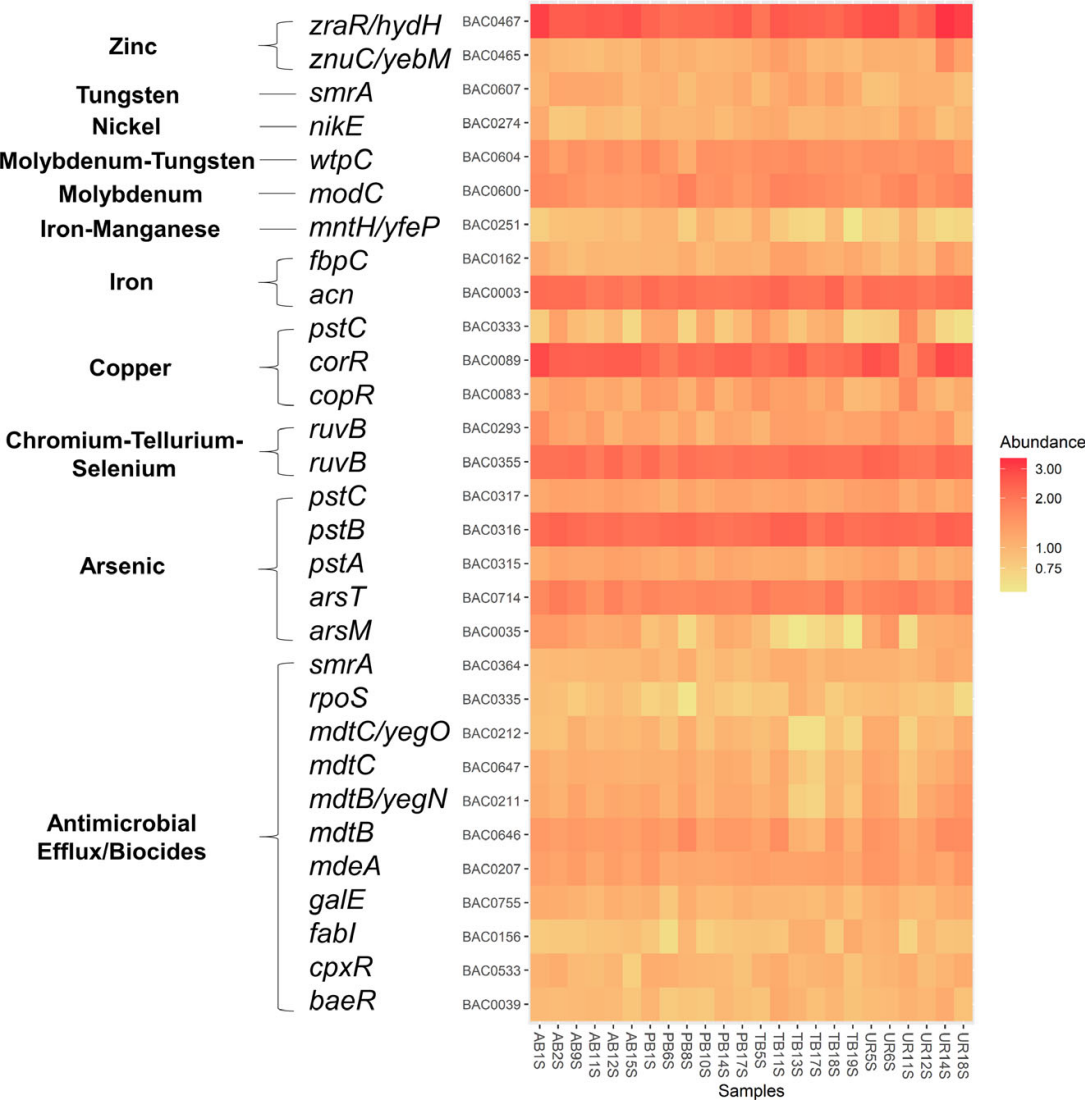
Heat map of variations of AB‐MRG‐like contigs contained in the top 30 AB‐MRG types. Data is based on the relative abundance of blast hits (BacMet v.2.0) for each respective sample.

Using the BacMet v2.0 database, we identified a large collection of genes associated with antibiotic, biocide and metal resistance (Table S13). Although reference genes are skewed more to MRGs, we found, similar to the analysis on ARGs using the SARGfam v.2.0 database, that the predominant ARG subtype included several resistance genes in the antimicrobial/biocide efflux category (36%) such as *mdtB*, *mdeA*, *cpxR*, *smrA*, *baeR* and *galE* (Figs 8 and 9, Table S14). The relative abundance of antimicrobial/biocide efflux varied and was higher on average than the reference site (2.36 genes per 16S rRNA copy) for both the Ash Basins (2.52 genes per 16S rRNA copy) and Pond B (2.54 genes per 16S rRNA copy; Table S15). There were no significant differences between numbers of antimicrobial/biocide resistance genes per 16S rRNA copy when comparing sites. When examining ARG subtypes, Shannon indexes ranged from 4.33 and 4.50, and we observed a significantly higher diversity of antimicrobial/biocide efflux resistance genes at Tim’s Branch compared to the reference site (*P* < 0.05; Fig. 7B, Table S16).

The other top resistance types included MRGs that occur primarily via complexes such as two‐component regulatory systems and ATP‐binding cassette (ABC) transporters (Figs 8 and 9, Table S15). These abundant MRG types included those that confer resistance to Cu (*corR*, *copR*, *ricR*; 11%), As (*pstB*, *arsT*, *pstC*; 10%), Fe (*acn*, *furA*, *bfrA*; 6%), Ni (*hoxN*, *nikE*, *nikD*; 5%), zinc (*zraR*/*hydH*, *znuC*/*yebM*, *troB*; 5%) and molybdenum (*modC*, *modB*, *modA*; 3%;Table S10). For example, the *zraR*/*hydH* gene, the most abundant MRG type overall in all samples (average 0.16 MRG genes per 16S rRNA copy), encodes a membrane‐associated protein kinase that is upregulated in response to high concentrations of Zn or Pb (Table S13). In addition, we found significant differences in MRG genes per 16S rRNA copy between the soil sites for several MRG types including As, Cu‐Ni‐Fe, Co‐gold and Fe‐manganese. With respect to the reference site, only Tim’s Branch had significantly higher genes for the MRG type of Cu‐Ni‐Fe (*P* < 0.05). In terms of MRG subtypes, Shannon indexes ranged from 4.29 to 4.60, and a significantly higher diversity of MRGs was observed in samples from Tim’s Branch compared to the reference site (*P* < 0.05; Fig. 7C; Table S17).

### Soil bacterial hosts of antibiotics, biocides and metal resistance genes

**Fig. 10:**
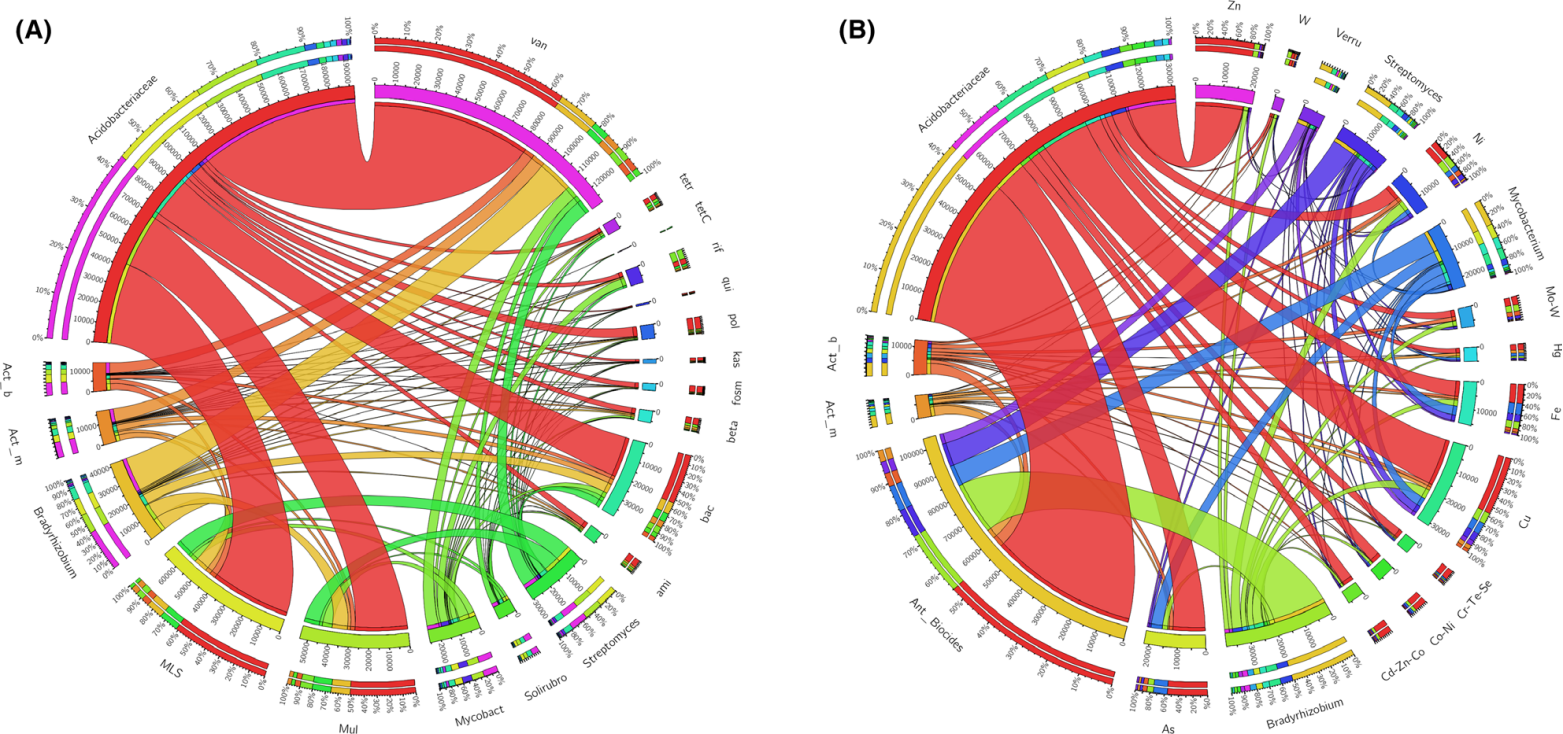
Circos plots displaying percentages of the top ARG‐like and MRG‐like carrying bacterial hosts. Bars surrounding plot represent the percentage of a particular ARG or MRG‐like gene that was observed in the bacterial hosts. Plots are based on the TPM data of reads mapped to (A) Sargfam and (B) BacMet v.2.0 database. Abbreviations are as follows: Act_b (Actinobacteria), Act_m (Actinomadura), Mycobact (Mycobacterium), Solirubro (Solirubrobacterales), MDR (multidrug), Tet (tetracycline), Amg (aminoglycoside), MLS (macrolide‐lincosamide‐streptogramin), Beta (β‐lactam), Bcr (bacitracin), Fos (fosmidomycin), Kas (kasugamycin), Pol (polymyxin), Qns (quinolone), Rif (rifamycin), Tcm (tetracenomycin), Van (vancomycin); Ant_Biocides (Antimicrobial Efflux/Biocides), Verru (Verrucomicrobia).

From the ARG‐like ORFs, we detected a myriad of diverse taxa, several of which formed a core resistome across all soil habitats. These included bacterial hosts such as Acidobacteriaceae, *Bradyrhizobium*, *Mycobacterium*, *Streptomyces*, Verrumicrobium, *Actinomadura*, Solirubacterales and several unclassified Actinobacteria (Fig. 10). Although the numbers of ARG‐like ORFs varied by site, the predominant bacterial host (19%) at the level of family or lower included several unclassified taxa in Acidobacteriaceae, which contained the majority of ARGs including genes that confer resistance to: vancomycin (42%), macrolide‐lincosamide‐streptogramin (MLS; 22%), multidrug (14%), bacitracin (11%), polymyxin (3%), beta‐lactams (2%), fosmidomycin (1%), aminoglycoside (1%) and others (3%). *Mycobacterium*, the second largest ARG‐like ORF containing host (5%), possessed resistance genes for: vancomycin (23%), multidrug (19%), rifamycin (16%), MLS (14%), bacitracin (9%), polymyxin (4%) and several others (15%; Fig. 10). *Streptomyces*, the third largest bacterial host (5%), possessed ARG‐like ORFs predominated by multidrug (27%), MLS (26%) and vancomycin (22%) resistance genes.

We detected similar patterns between taxa with respect to antimicrobial/biocide and metal resistance ORFs with the major bacterial hosts including Acidobacteriaceae (27%), *Bradyrhizobium* (7%), *Mycobacterium* (4%) and *Streptomyces* (3%; Fig. 10). Several unclassified genera in the family Acidobacteriaceae possessed resistance genes for antimicrobial/biocide efflux (44%), Cu (12%), Zn (12%), As (9%), Fe (5%), Ni (5%), cobalt‐nickel (3%) and several others. Taxa in the genus *Bradyrhizobium* possessed resistance genes for antimicrobial/biocide efflux (51%), Cu (9%), Zn (8%), As (7%), Fe (5%), Ni (5%), cobalt‐nickel (4%). *Mycobacterium* possessed resistance genes for antimicrobial/biocide efflux (34%), Cu (25%), Zn (12%), As (11%), Fe (6%) and Ni (3%). Finally, *Streptomyces* possessed resistance genes in similar proportions to Acidobacteriaceae and *Bradyrhizobium* with antimicrobial/biocide efflux the largest in AB‐MRG (46%), followed by Cu (13%), Zn (11%), As (8%), Fe (4%), among several others.

### Co‐occurrence of antimicrobial/biocide efflux and metal resistance genes in SRS soils

**Fig. 11:**
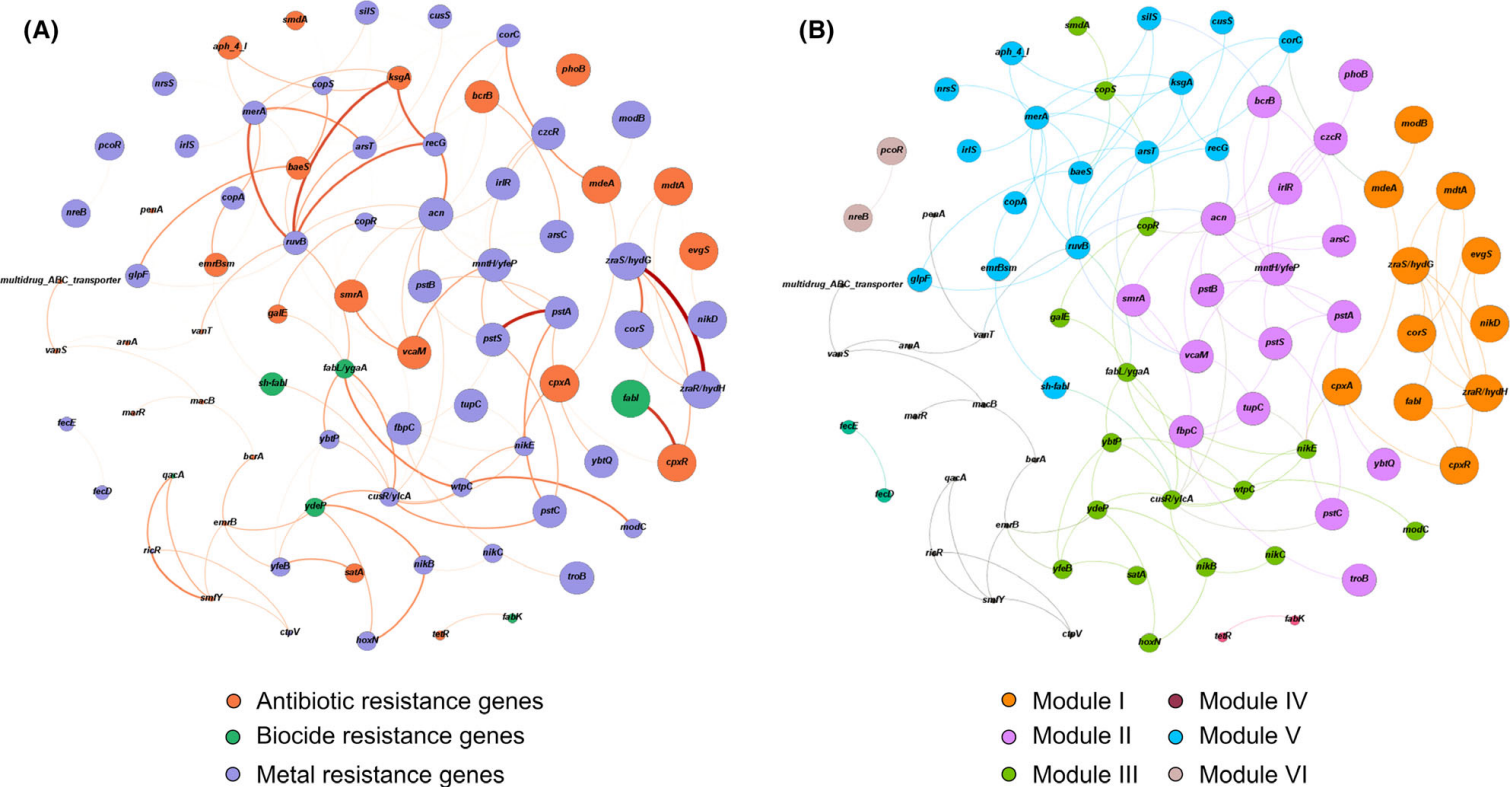
The network analysis showing the co‐occurrence patterns of antibiotic, biocide and metal resistance genes detected in the top 24 taxa. A. The nodes with different colours represent antibiotic (orange), biocide (green) and metal resistance genes (light purple). The intensity of edges corresponds to the degree of the positive correlations ranging from 0.61 (light orange) to 0.91 (dark red). B. The nodes with different colours represent the six modularity classes, with the colours of edges corresponding to their respective class: module I (orange), module II (pink), module III (green), module IV (dark red), module V (cyan) and module VI (light brown). A connection represents a strong spearman correlation (*P* > 0.6) and significant (*P* < 0.05) correlation (FDR). The size of each node is proportional to the number of connections.

We focused primarily on the predominant ARG‐like and MRG‐like ORF containing taxa (*n* = 24) across all soil habitats. Seven modules were identified based on the resistance subtypes (Fig. 11A and B). The largest module (module VI), contained 17 subtypes, the majority consisting of genes conferring multi‐resistance to As, Co, Cd and Zn (e.g., *pstA*, *pstB*, *pstC*, *arsC*, *irlR*, *czcR*) with the metal membrane transporter *mntH/yfep* acting as the hub (i.e., a node with large number of connections). Module IV, in which *merA* was the hub, consisted of 16 subtypes, including genes conferring resistance to aminoglycosides (*aph4_I*, *ksgA*) and non‐metals/metals such as selenium, tellurium, silver, Ni and Cu (*recG*, *baeS*, *silS*, *cusS*, *corC*, *copA*). Module III, in which *cusR/ylca* was the hub, also contained 16 subtypes, including several metal resistance genes for Cu, Fe, Ni and molybdenum (*copR*, *copS*, *yfeB*, *nikB*, nikC,) and biocide resistance (*galE*, *fabL/ygaA*, *ydeP*). All subtypes in the network co‐occurred (O% = 76%) more than would be expected by chance (R% = 0.03%). A co‐occurrence network including all the soil taxa annotated to the ARG or MRG‐like ORFs displayed non‐random co‐occurrence patterns between all subtypes (O% = 0.004%, R% = 0.01%; Table S18).

## Discussion

In this study, we used microbiome and metagenomic analyses to examine microbial communities and associated patterns of antimicrobial and metal resistance in soils from the Savannah River Site, a historic area with legacy contaminants including heavy metals and radionuclides. Although contamination is distributed non‐uniformly, previous studies have indicated that certain areas (e.g., Ash Basins) are heavily enriched with a range of heavy metals (e.g., aluminium, arsenic, cadmium, copper, iron, mercury, manganese, nickel, selenium and zinc) and can be present at levels significantly higher than those found at reference locations (Tannenbaum and Beasley, 2016). Moreover, numerous studies conducted at this site spanning over two decades have provided both experimental and observational evidence that suggests elevated heavy metal contamination can function as an indirect selective agent for the acquisition of antibiotic resistance in bacteria (Leff *et al.*, 1993; Stepanauskas *et al.*, 2006; Wright *et al.*, 2006; McArthur *et al.*, 2016; Agarwal *et al.*, 2019). These studies have shown that bacteria can possess genes that provide indiscriminate tolerance to both heavy metals and antibiotics (cross‐resistance), and/or co‐resistance whereby two or more antimicrobial resistance genes are genetically linked on the same mobile genetic element (Baker‐Austin *et al.*, 2006; Seiler and Berendonk, 2012). Furthermore, increased levels of heavy metals in the environment have been shown to co‐regulate genes responsible to antibiotic resistance and decrease antibiotic susceptibility (Baker‐Austin *et al.*, 2006; Seiler and Berendonk, 2012). Considering the above, the co‐selection phenomenon represents a collection of clinically important mechanisms that can contribute to the dissemination and maintenance of antibiotic resistance, and thereby poses a significant public health concern.

Results from trace metals analysis indicated that there were no significant differences in the soil concentrations for several metals, however, significantly higher concentrations of Cobalt and Strontium were observed in soils from Ash Basins and Tim’s Branch. Given the similarly in the other trace metal concentrations among the four sites, we expected the structure and diversity of the soil microbial communities to be similar, but this was not the case. Instead, we observed that while soil communities were similarly structured, being predominated by Proteobacteria (27.24% ± 0.02%), Acidobacteria (22.00% ± 0.04%) and Actinobacteria (14.83% ± 0.02%), among others, there was a higher abundance and diversity of taxa in samples from the reference site (Upper Three Runs) compared with samples from Ash Basins, Tim’s Branch and Pond B. Many studies on soils have demonstrated that high levels of heavy metal contamination can significantly decrease microbial diversity and biomass (Moffett *et al.*, 2003; Chen *et al.*, 2014, 2019; Quadros *et al.*, 2016). Others have shown that long‐term exposure to heavy metals can lead to increases in bacterial diversity due to the increase in other edaphic parameters that promote the bacterial community during the course of remediation (Bourceret *et al.*, 2016). Thus, it was not entirely surprising that certain taxa from the sites with legacy contamination displayed significantly higher (*P* < 0.05) abundances of several bacterial orders including Rhodospirillales (Alphaproteobacteria), Solirubrobacterales (Actinobacteria), Rhizobiales (Alphaproteobacteria), Pseudomanadales (Gammaproteobacteria), Burkholderiales (Betaproteobacteria) and Saprospirales (Bacteroidetes). To explain these differences, we examined the beta‐diversity of the soil habitats to illuminate the compositional dissimilarity between communities.

Our DistLM and dbRDA analysis revealed strong correlations between edaphic properties and the metal concentrations with the bacterial communities present in the soils. Previous studies on soils suggest that pH is one of the primary predictors of bacterial community structure (Fierer and Jackson, 2006; Lauber *et al.*, 2009; Wu *et al.*, 2013). However, despite significant differences in soil pH between the four sites, other edaphic factors such as soil water content and the presence of heavy metals appeared to be stronger drivers of bacterial community structure, even for the reference site. It should be noted that although the results were significant and supported our hypothesis (i.e., HMs would predict the structure of bacterial communities) the data should be interpreted with caution. For instance, despite the fitted model in the dbRDA plot displaying a large percentage of explained variation in the soil using the edaphic properties and HMs data provided, the total per cent variation explained was very low. This suggests that there are confounding factors not measured in this study which could also contribute to soil bacterial community structure and diversity. Additional factors known to influence the communities includes soil depth, soil type, salinity, porosity, sulphur, nitrogen, trace minerals, temperature, dead organic matter and presence of fungi and plants (Wieland *et al.*, 2001; Zhou *et al.*, 2002; Guan *et al.*, 2013; Dotaniya and Meena, 2015; Kaiser *et al.*, 2016).

Recent advances in next‐generation sequencing (NGS) have enabled an unprecedented view into complex microbial communities and the search for non‐cultivable producers of antimicrobial resistance genes (Asante and Sekyere, 2019). Shotgun metagenomics allowed us to analyse soil microbial communities *de novo* without prior knowledge of the thousands of currently known ARGs and their homologs necessary for primer design (Asante and Sekyere, 2019). Our metagenomic analyses revealed a large collection of genes associated with resistance to antibiotics, biocides and heavy metals harboured by a myriad of diverse non‐cultivable taxa. Relative abundances of ARGs in soils ranged from 0.39 to 0.46 genes per 16S rRNA copy, with the most common soil ARG types detected including genes that confer resistance to multidrug (52%), vancomycin (28%) and fosmidomycin (5%). The relatively high abundance of multidrug resistance genes was expected, as several studies have demonstrated their ubiquity in bacteria from a range of environments (Chen *et al.*, 2014, 2019; McArthur *et al.*, 2016; Hu *et al.*, 2017). Multidrug efflux pumps are typically chromosomally encoded and are conserved across the majority, if not all, strains of bacterial species (Blanco *et al.*, 2016). In addition to possessing the capacity to effectively reduce the concentration of antibiotics, they can also extrude a wide range of environmental insults including heavy metals, organic pollutants, plant‐produced compounds, foreign metabolites, among others (Blanco *et al.*, 2016). A previous study indicated that many of these RND efflux pumps can be categorized into four functional subfamilies, of which two were found to be involved in the extrusion of antibiotic (group 1) and heavy metals (group 3) antibiotics, respectively (Godoy *et al.*, 2010).

Outside of the multidrug ARG type, the relative abundance of the predominant ARGs in this study differed from several others on soils in which ARGs such as β‐lactamases, macrolide‐lincosamide‐steptogramin, aminoglycoside were also frequently occurring (Forsberg *et al.*, 2014; Hu *et al.*, 2016, 2017; Gou *et al.*, 2018). Notably, our results were particularly interesting with respect to vancomycin, a ‘last resort’ broad‐spectrum antibiotic rarely used in hospital settings. Vancomycin resistance genes have been detected in bacterial genomes from a wide range of environments including soils, oceans, human faeces and even ancient 30,000‐year‐old permafrost with no evidence of human contact (D'Costa *et al.*, 2011; Nesme and Simonet, 2015). Considering the soil environment is a highly diverse reservoir of bacteria and their gene products, the *arms‐shield* hypothesis suggests that the diversity of resistance genes for vancomycin and other antibiotics in soils can be explained by the continuous exposure to inhibitory concentrations of antibiotics secreted by competing microorganisms (Nesme and Simonet, 2015). In fact, it was proposed that *van* operons observed in clinically relevant human–pathogens (e.g., *Enterococcus faecium*) originated from glycopeptide‐producing bacteria residing in the soil, and thus likely evolved from a common ancestor based on analyses of sequence homology (Guardabassi *et al.*, 2005). We further examined *van* clusters detected in our dataset using publicly available RefSeq proteins on https://www.ncbi.nlm.nih.gov/. We blasted these vancomycin resistance genes against Acidobacteria (our predominant taxa) genomes and found that they have low similarity (≤ 45% predicted amino acid identity) compared to enterococcal and *vanA*/*vanB* operons, however, sequence homology increased to ≥ 77% for other common soil bacteria (Actinomycetales, *Streptomyces* spp., *Rhodococcus* spp.; Arthur and Quintiliani, 2001; Guardabassi *et al.*, 2005; data not shown). These results suggest that the high prevalence and ubiquity of vancomycin resistance genes in the SRS soils may enhance survival and fitness; however, it would be difficult to test this without further experiments.

Furthermore, although a previous global survey of environmental metagenomes concluded that vancomycin resistance genes were common in a myriad of environments, to our knowledge none of these environments possessed relative abundances comparable to SRS soils (Nesme *et al.*, 2014; Nesme and Simonet, 2015). This was true even for the reference site (30% of total Upper Three Runs ARGs), suggesting that vancomycin resistance genes are abundant and ubiquitously present at the Savannah River Site. However, we did see lower abundances relative to the reference site in ARG types such as β‐lactamases (Ash Basins), rifamycin (Tim’s Branch), tetracycline (Tim’s Branch) and fosfomycin (Tim’s Branch). Considering this, we examined the diversity of ARG subtypes and discovered that there were significant differences, with a greater number of ARG subtypes (including biocides) observed in soils from Tim’s Branch than in soils from the reference site. These results indicate that human activities may play an important role in the dissemination and enrichment of antibiotic resistance genes in the environment. A previous study on soils indicated that several ARG types were positively correlated with soil copper levels, whereas chromium, nickel, lead and iron correlated with specific ARG types (Knapp *et al.*, 2011).

In examining MRG types, we found that SRS soils contained a diverse collection of genes associated with copper, arsenic, iron, nickel, zinc, molybdenum resistance and multi‐metal resistance. The zinc resistance gene *zraR*/*hydH* was the most abundant MRG subtype, and this along with copper resistance genes have been reported in the literature as being strong contributors to resistance traits, having direct correlations with as β‐lactamases, sulphonamides, macrolide‐lincosamide‐streptogramin and tetracycline resistance genes (Knapp *et al.*, 2017). Arsenic associated resistance genes such as those in the *ars* gene clusters have been shown to be globally distributed and are suggested to have prominent roles in global arsenic biogeochemistry (Ben Fekih *et al.*, 2018; Dunivin *et al.*, 2019; Firrincieli *et al.*, 2019). Iron and nickel resistance genes have also been shown to be significantly associated with ARGs such as multidrug, β‐lactamases, sulphonamides, macrolide‐lincosamide‐streptogramin, tetracycline, aminoglycosides and vancomycin resistance genes (Guo *et al.*, 2014; Hu *et al.*, 2017). Our study aligns with observations of MRGs reported elsewhere and suggests that their wide occurrence is due to intrinsic properties of the soil environment in addition to anthropogenic activities (e.g., medical waste, agriculture, industrial activities, etc.).

Finally, we demonstrated significant correlations between a variety of antibiotics, biocides and metal resistance genes in the soil environments at SRS. For certain dominant MRG subtypes with unclear antimicrobial associations, such as those involved in molybdenum transport (ModABC complex), we found significant correlations with antimicrobial/biocide efflux (*mdeA*, *fabgL*/*ygaA*) not reported elsewhere. This was particularly helpful in understanding the co‐occurrence network for our particular study site. Other co‐occurrence patterns were largely in agreement with a myriad of studies that have also demonstrated co‐selection of antibiotic resistance from metals (Seiler and Berendonk, 2012; Wales and Davies, 2015; Cesare *et al.*, 2016) and; we found that these patterns were often non‐random, which although not a new phenomenon, further illustrates that even low total metal levels can affect ARG abundance in soils (Knapp *et al.*, 2011). Given the long‐documented history of metal and radionuclide contamination at the Savannah River Site, and the global concerns over the spread of increasingly resistant bacteria, our study is particularly relevant in understanding, and ultimately reducing the risk of antimicrobial resistance.

## Conclusion

Soils are heterogeneous habitats that possess bacterial communities with considerable genetic diversity at very small spatial scales. This soil ‘resistome’ containing the gene products of millions of bacteria is speculated to be a large source of resistance gene determinants. Our study demonstrated strong associations between the microbial community in soils at the SRS, and the presence of chronic heavy metal and radionuclide contamination. We found that certain bacterial taxa are differentially affected by edaphic factors and heavy metal contamination and that their presence can have an important role in the dissemination and maintenance of antimicrobial and metal resistance. The relative abundance and diversity of ARGs and MRGs was significantly higher at Tim’s Branch, a site with historic heavy metal and radionuclide contamination, compared to the reference site. However, our results suggest that even in areas with low levels of heavy metals, antibiotic and heavy metal resistance can be both ubiquitous and widespread.

## Experimental procedures

### Study areas

In this study, we collected soils from four locations with habitats consisting of mixed hardwood and pine forest; three contaminated sites, and a reference site located on the SRS (Fig. 1; Tannenbaum and Beasley, 2016). The contaminated sites included an area with elevated metal and metalloid concentrations (D‐area coal‐fly ash basin; Ash Basins), an area with only radionuclide contamination (Pond B), and an area with both metals and radionuclide contamination (Tim’s Branch). Soils from Ash Basins are contaminated with coal combustion waste containing a range of metals and metalloids such as arsenic (As), chromium (Cr), cadmium (Cd), copper (Cu), iron (Fe), nickel (Ni), selenium (Se) and Zinc (Zn; Tannenbaum and Beasley, 2016). Pond B is a reservoir that was originally constructed as a secondary cooling system for nuclear production at R Reactor until it was decommissioned in 1964 (McCreedy *et al.*, 1997; Garten *et al.*, 2000). Between 1961 and 1964, R Reactor discharged several radionuclides (e.g., ^3^H, ^137^Cs, ^90^Sr, americium‐241, cerium‐244 and plutonium‐239, 240) into Pond B as effluents. Tim’s Branch is a second‐order stream that receives contamination from an eroding former discharge settling pond, Steed Pond. Steed Pond received up to 44 000 kg of depleted and natural uranium (U), as well as similar quantities of Ni from an aluminium‐clad nuclear reactor, between 1954 and 1985 (Murray *et al.*, 2010). Tim’s Branch soils are contaminated with aluminium (Al), Cu, Cr, Zn, lead (Pb) and depleted U (Tannenbaum and Beasley, 2016). Upper Three Runs Creek (UR), a 40‐km waterway that empties into the Savannah River, was used as the reference location in this study. It is the only major tributary at SRS that has not received any thermal, chemical or radioactive pollutants (Murray *et al.*, 2010).

### Sample collection and processing

Briefly, 15 g of topsoil (0–20 cm depth) samples were collected near GPS points previously used in a study conducted by Tannenbaum and Beasley (2016) using a 70% ethanol‐sterilized garden shovel. Soil collection was staggered, with collections taking place within 10–15 m of contaminated water bodies. All 80 samples (20 samples per site) were placed on ice until eventual storage at −20°C. Soils were composited by site and subsampled before analysis (10 subsamples per site). Soil heavy metals analysis was conducted using the EPA Method 3052 with modifications (Wu *et al.*, 1996). All digestions were analysed for extractable As, Co, Cu, Cr, Ni, strontium (Sr), U and Zn, using an inductively coupled plasma mass spectrometer (ICP‐MS). Edaphic factors such as total phosphorus (P), total carbon (C), total nitrogen (N) and pH were analysed using modified protocols developed by the University of Georgia Stable Isotope Ecology Laboratory (https://cais.uga.edu/siel-soil-analysis/). Soil total phosphorus, was conducted using acid–persulphate digestion (Nelson, 1987). Micro‐Dumas combustion analysis was used for total carbon and total nitrogen analysis (Hauck, 1982; Kirsten and Hesselius, 1983). Soil pH was measured using a pH analyser placed in a 1:1 mixture of soil and a 0.01 M calcium chloride solution. We used linear regression to compare metals present in the soil among different sites using JMP Pro 13 (SAS, Cary, NC, USA). Trace elements were log‐transformed, when necessary, to meet assumptions of normality. Post hoc multiple comparisons were made using t‐tests to further examine site differences in trace elements.

### DNA isolation, PCR amplification and Illumina Sequencing of 16S rRNA

DNA was extracted from 0.5 g of soil using a MoBio PowerSoil DNA isolation kit (MoBio, Carlsbad, CA, 92010, USA) and purified by a magnetic‐based size selection method using SeraPure Speedbeads (Thermo‐Fisher Scientific, Asheville, NC, 28804, USA) according to the manufacturer’s protocol. PCR libraries for bacterial 16S were generated using the S‐D‐Bact‐041‐b‐S‐17 (5′‐CCTACGGGNGGCWGCAG‐3′) forward and S‐D‐Bact‐0785‐a‐A‐21 (5′‐GACTACHVGGGTATCTAATCC‐3′) reverse primer pair, which amplify an approximately 444 bp fragment (Klindworth *et al.*, 2013). Modifications to the primer sets (fusions: 8 forward + 12 reverse) were done according to TaggiMatrix protocol which included fusions with universal 5’ iTru sequences containing up to 96 dual combinations of unique internal tags (Glenn *et al.*, 2019b). DNA from each sample was PCR amplified using the 16S‐iTru fusions in 25 μl reactions using the KAPA HiFi Hotstart PCR kit (KAPA Biosystems, Wilmington, MA, 01887, USA). The PCR amplification protocol was as follows: 95°C for 3 min, followed by 30 cycles of 95°C for 1 min, 55°C for 30 s and 72°C for 30 s with a final elongation step of 72°C for 5 min. Amplicons were visualized using 1.5 % agarose gel, pooled, and then purified with SeraPure Speedbeads. Using the initial PCR products as a template, we conducted a second round of PCR with dual‐indexed iTru primers to construct full‐length Illumina libraries. All PCR reactions were performed using an T100 Thermal Cycler (BioRad, Hercules, CA, 945477, USA; Glenn *et al.*, 2019a). The 16S libraries were combined with iTru library pools from other experiments using a single run Illumina MiSeq v2 500 cycle kit (PE250) at the Georgia Genomics and Bioinformatics Core (GGBC, University of Georgia).

### Demultiplexing and quality filtering

All fastq conversion and demultiplexing based on outer indexes (iTrus) was conducted using bcl2fastq (Illumina, v1.8.4) and the Fastx toolkit v.0.0.14 (http://hannonlab.cshl.edu/fastx_toolkit/links.html) was used to remove low quality reads. Paired‐end sequencing reads were imported into Geneious v.10.0 (Biomatters Limited, NJ, USA), set as paired‐reads with an insert size of 444 bp and trimmed to remove Illumina adapters using default settings. Paired‐end sequencing reads were then merged using FLASH v.1.2.9 plugin (Magoč and Salzberg, 2011). The software package Mr_Demuxy v1.2.0 (https://pypi.python.org/pypi/Mr_Demuxy/1.2.0 ) was utilized to demultiplex the merged reads into individual combinatorically tagged fastq files based on internal indexes (barcodes). Sequences were filtered based on size (≥ 400 bp) and quality scores (≥ Q20).

### Soil Bacterial 16S rDNA sequence analysis

Sequencing reads were imported into the phylogenetic software package MacQiime v1.91 (http://www.wernerlab.org/software/macqiime) for Operational Taxonomic Unit (OTU) identification, multi‐levelled taxonomic classification and diversity estimates (Caporaso *et al.*, 2010). All de‐multiplexed raw 16S rDNA sequences are available through NCBI under BioProject PRJNA616017. Briefly, samples were sorted and bacterial OTUs were selected using the Greengenes v.13_8 16S rRNA gene reference database and the VSEARCH OTU picking strategy (DeSantis *et al.*, 2006; Rognes *et al.*, 2016). Taxonomy was defined using default settings of ≥ 97% similarity to reference sequences. In addition, we also utilized deblurring, a sub‐operational‐taxonomic approach (sOTU) to Illumina‐based 16S amplicon sequencing. Deblur uses error profiles to denoise Illumina data and is capable of differentiating amplicons by a single base pair. Deblur was used to perform a higher resolution inspection of the bacterial community (Amir *et al.*, 2017).

Within‐sample (alpha) diversity was evaluated in QIIME v1.91 and visualized with the Phyloseq v.3.10 package using various metrics (Chao1, ACE, Fisher’s Alpha, Shannon‐Wiener, and Simpson; McMurdie and Holmes, 2013). Alpha diversity of communities found in soil samples was computed using a sequencing depth set to 15 000 sequences. To test for the significant variation in taxonomic richness across the four sampling sites, we used the non‐parametric Kruskal–Wallis test with the False Discovery Rate (FDR) correction (Kruskal, 1964; Storey, 2011). We used QIIME v.1.91 to test for the significant variation in the frequency of individual OTUs across the four sites, using Kruskal–Wallis test with FDR correction for multiple comparisons, and the Monte Carlo simulated non‐parametric *t*‐test for pair‐wise comparisons (Kruskal, 1964; Ortmann and Lu, 2015). Bray–Curtis distances between samples (beta‐diversity) were computed and visualized using PRIMER 7.0 software with PERMANOVA + add‐on (Primer‐E, United Kingdom) as several distance‐based plots (Bray and Curtis, 1957). These included several distance‐based analyses described in previous studies such as a non‐metric multi‐dimensional scaling plot (NMDS), canonical analysis of principle coordinates (CAP) for testing our *a priori* hypotheses (e.g., site predicts soil microbiota), and distance‐based redundancy analysis (dbRDA) to discern the direction and magnitude of the relationship between edaphic properties and heavy metals with respect to soil microbiota (Kim *et al.*, 2015; Paliy and Shankar, 2016). To identify the key drivers of soil bacterial community structure for the soils collected from the four sites at the SRS, we also performed a distance‐based linear model (DistLM), which is an extension of distance‐based redundancy analysis (dbRDA; Legendre and Anderson, 1999).

### Screening of 16S rDNA sequences with PICRUSt and construction of metagenomic library

The software PICRUSt was used to make gene content predictions based on the 16S rRNA gene sequences using the Greengenes v. 13_5 as a reference database (Langille *et al.*, 2013). A total of 37 antibiotic and metal resistance KEGG orthologs (KOs) were selected by manually searching the KEGG database. KO functional profiles were examined using Statistical Analysis of Metagenomic Profiles (STAMP; Parks *et al.*, 2014). We used PICRUSt results to assign samples in three categories based on the expected resistance categories: ‘Low’, ‘Medium’ and ‘High’. These categories encompassed a percentage range of 0.05% to 0.15% corresponding to the proportion of genes detected relative to number of 16S rRNA sequences. For each category, we selected two samples, for a total of six samples per collection site selected (Ash Basins, Tim's Branch, Pond B and Upper Three Runs) for a total of 24 samples. Metagenomic libraries for these samples were prepared using NEB Ultra II FS kits (New England Biolabs, Ipswich, MA, USA) following manufacturers protocol at half volume reactions with two modifications. We used 5 μM iTru y‐yolk adaptors during ligation and 5 μM of iTru indexed primers during PCR for 9 cycles (Glenn *et al.*, 2019a). Resulting libraries were verified on a 1.5 % agarose gel, cleaned with SeraPure Speedbeads, quantified using a Qubit 2.0, and pooled for sequencing on a HiSeq 3000 PE150. The PCR thermocycler conditions consisted of the following: 98°C 30 s, then 9 cycles of 98°C 10 s, 65°C for 1:15 min, then 65°C for 5 min, 4 min hold. All raw metagenomic sequences are available through NCBI under BioProject PRJNA616018.

### Calculations of ARG and MRG abundance

Abundance of ARGs from the PICRUSt pre‐screened samples were determined using the ARGs‐OAP v.2.0 pipeline (Yin *et al.*, 2018). Briefly, the fastq reads were quality trimmed to obtain clean reads, ARG reads and 16S rRNA genes were extracted from all the samples, then ARG reads were identified and annotated using a BLASTX approach by searching the combined ARG databases of CARD (The Comprehensive Antibiotic Resistance Database) and ResFinder (Zankari *et al.*, 2012; McArthur *et al.*, 2013). The ARG reads were normalized by the number of 16S rRNA copies detected, and ARG abundance was expressed as of ARGs per copy of 16S.

Abundance of antimicrobial/biocide efflux genes (ARGs) and metal resistance genes (MRGs), collectively AB‐MRGs was determined using a hybrid approach method described in a previous study (Yang *et al.*, 2014). Using several custom scripts and the paired‐end reads from the 16S libraries, candidate reads were initially identified through UBLAST using a cut‐off *E*‐value of 10^−7^, sequence identity of 90% and alignment length greater than 25 amino acids (Supplemental Methods). Reads were annotated by applying the BacMet (version 2.0) antibacterial biocide and metal resistance genes database and using 753 experimentally confirmed genes (as of March 2018; Pal *et al.*, 2014). Matched sequences were extracted, and further annotation was performed using BLASTX for more accurate annotation using the same cut‐off *E*‐value, sequence identity and alignment length as stated above. The abundance of reads was calculated using the equation described by Li *et al.* (2015) and the number of 16S rRNA copies originally detected by the ARGs‐OAP v.2.0 pipeline. The abundance was expressed as number of ARGs or MRGs per copy of 16S. It should be noted that several of the MRG types in the BacMet v.2.0 database encode regulatory proteins that respond to multiple metals, we categorized these genes into groups (e.g., ‘Copper’, ‘Copper–Silver’ and ‘Copper–Zinc’).

### Metagenome assembly and identification of ARG and MRG‐like open‐reading frames (ORFs)

High‐quality reads for the 24 selected samples were de novo assembled using the default *k*‐mer size using Spades v.3.9.0 (Bankevich *et al.*, 2012). The software Prodigal v.2.60 was used to predict ORFs within contigs (Hyatt *et al.*, 2010). We conducted an HMMSCAN (http://hmmer.org) against the SARGfam database (https://github.com/xiaole99/SARGfam) using the ORFs as input sequences and validated HMM profiles of antibiotic resistance genes (Yin *et al.*, 2018). The ARG‐like ORFs from the HMM results were extracted from the original protein sequences of ORFs predicted in the contigs using Seqkit v0.12.0 (https://bioinf.shenwei.me/seqkit/usage/). PROKKA v.1.14 was also used to functionally annotate the predicted ORFs, using the SARGfam HMM profile as input (Seemann, 2014). Similarly, the MRG‐like ORFs were first annotated using BLASTP against BacMet v.2.0 database at an *E*‐value ≤ 10^−5^. The results were used to extract candidate sequences, which were further annotated in PROKKA, using the BacMet v.2.0 protein database containing 735 experimentally confirmed resistance genes.

### Mapping reads and determining ARG and MRG‐like ORF coverage

Bowtie2 v.2.3.5.1 was used to map all clean sequencing reads to the extracted ARG‐like ORF nucleotide sequences of each respective sample group. SAMtools (http://samtools.sourceforge.net/) was used to convert the SAM files to BAM format, and sort by alignment coordinate. Reads that mapped to the assembly, were counted using the prokkagff2gtf and htseq‐count scripts (Anders *et al.*, 2015). Abundance values for genes were normalized to predicted transcripts per million (TPM) using the tpm_tably.py, which calculates the TPM based on average read length and length of gene. Further details are described in a previous study by Anders *et al.* (2015).

### Taxonomic assignment of bacterial hosts of ARGs and MRGs

Diamond v.0.9.30 was used to annotate the ORFs, by conducting a BLASTP search against the NCBI NR database (downloaded on May 29, 2019) at *E*‐value ≤ 10^− 5^ (Buchfink *et al.*, 2015). The diamond BLASTP results were ‘meganized’ and annotated using MEGAN (MEtaGenome ANalyzer, Version 6.16.4) taxonomic assignment, using default parameters (voting score of ≤ 50%; Huson *et al.*, 2007). Major bacterial hosts of ARGs and MRGs shared between sites were visualized using Circos Table Viewer v0.63‐9 (Krzywinski *et al.*, 2009).

### Network of co‐occurrence analysis of antibiotic, biocide and metal resistance genes

Correlation‐based network analysis was performed by generating a sparse matrix based on the combined TPM data from both the SARGfam and BacMet database. This matrix was filtered based on the top 25 most abundant taxa and a co‐occurrence network was generated using custom scripts (https://github.com/RichieJu520/Co-occurrence_Network_Analysis) detailed in a previous study (Ju *et al.*, 2014). The resulting co‐occurrence network was visualized in Gephi v.0.9.3 (Bastian *et al.*, 2009).

### Statistical analysis

Differences in microbial community structure and composition between the four sampling sites were tested using permutational multivariate analysis of variance (PERMANOVA) on log‐transformed Bray–Curtis dissimilarity values with soil sampling location as a fixed effect (Anderson 2005). We used a type I partial sum of squares design and Monte Carlo sampling permutated 999 times over residuals under a full model. The R package v.3.6.3 was used to conduct one‐way analysis of variance (ANOVA) or Kruskal–Wallis one‐way analysis of variance, depending on pass or failure of a Shapiro–Wilk test of normality, to compare differences in relative abundance of ARG or MRG number per 16S rRNA copy, in addition to assessing differences in Shannon index. For network analyses, the observed (O%) and random incidences (R%) of co‐occurrence patterns between antibiotics, biocides and metals were determined following methods described in a previous analysis (Ju *et al.*, 2014, 2016; Yang *et al.*, 2019).

## Conflict of interest

The authors declare no conflicting interests.

## Disclaimer

This report was prepared as an account of work sponsored by an agency of the United States Government. Neither the United States Government, nor any agency thereof, nor any of their employees makes any warranty, express or implied or assumes any legal liability or responsibility for the accuracy, completeness or usefulness of any information, apparatus, product or process disclosed or represents that its use would not infringe privately owned rights. Reference herein to any specific commercial product, process or service by trade name, trademark, manufacturer or otherwise does not necessarily constitute or imply its endorsement, recommendation or favouring by the United States Government or any agency thereof. The views and opinions of authors expressed herein do not necessarily state or reflect those of the United States Government or any agency thereof.

## Supporting information


**Table S1.** Group differences in soil taxa.
**Table S2.** Group differences in soil taxa between Upper Three Runs and Ash Basins.
**Table S3.** Group differences in soil taxa between Upper Three Runs and Pond B.
**Table S4.** Group differences in soil taxa between Upper Three Runs and Tim's Branch.
**Table S5.** Effects of main factors and their interactions assesed by PERMANOVA. Main factors represent site (Location) (Ash Basins, Pond B, Tim's Branch, Upper Three Runs). Values in table represent the F‐ratio (F), the level of significance, ns, not significance (P < 0.05).
**Table S6.** Relative position of soil samples in the biplot is based on log transformed Bray Curtis dissimilarities. Vectors indicate the weight and direction of those edaphic properties or heavy metals that were best predictors of soil bacterial composition as suggested by the results of the distance‐based linear model (distLM). The dbRDA axes describe the percentage of the fitted or total variation explained by each axis while being constrained to account for group differences.
**Table S7.** PICRUSt predicted metagenome contributions of soil samples.
**Table S8.** Metagenome sequencing summary.
**Table S9.** Information on assembly, contigs and ORFs.
**Table S10.** ARG types by site (percentage).
**Table S11.** Relative abundance of ARGs per 16S copy. (*) indicates significant differences in ARG type based on one‐way ANOVA or Kruskal‐Wallis analysis of variance (P < 0.05).
**Table S12.** Alpha diversity indices for ARGs (SARGfam).
**Table S13.** Bacmet Ids, genes, and antibiotic, biocide, or metal type.
**Table S14.** Relative abundance of ARGs/MRG types detected using BacMet v2.0 database.
**Table S15.** Relative abundance of ARGs/MRGs per 16S copy (BacMet v2.0 database). (*) indicates significant differences in ARG type based on one‐way ANOVA or Kruskal‐Wallis analysis of variance (P < 0.05)Relative abundance of ARGs/MRGs per 16S copy (BacMet v2.0 database). (*) indicates significant differences in ARG type based on one‐way ANOVA or Kruskal‐Wallis analysis of variance (P < 0.05)
**Table S16.** Alpha diversity indices for antimicrobial/biocide efflux genes (BacMet).
**Table S17.** Alpha diversity indices for MRGs (BacMet).
**Table S18.** Obeserved vs Random Co‐occurrence.

**Data S1.**Supplementary Methods and scripts used for Ublast_blastx approach

## Data Availability

Sequence data has been provided in the main document under Bioprojects PRJNA616017 and PRJNA616018.
