# Supplementary material for: Co‐occurrence of antibiotic, biocide, and heavy metal resistance genes in bacteria from metal and radionuclide contaminated soils at the Savannah River Site
Source: Microb Biotechnol. 2020 May 3;13(4):1179–200. doi: 10.1111/1751-7915.13578 (PMC7264878; doi:10.1111/1751-7915.13578)
Supplement: Supplementary file 2 — Data S1.Supplementary Methods and scripts used for Ublast_blastx approach [file MBT2-13-1179-s002.docx]

**Supplementary Methods and scripts used for Ublast_blastx approach**

Methods based on original article by (Yang *et al.*, 2014)

**Quick search for candidate sequences from MRG database using USEARCH *ublast* command** (Edgar, 2010)**:**

ls *.fa | parallel –gnu “usearch -ublast {} -db “your_database.fasta” -evalue 1e-5 -accel 0.5 -blast6out {/.}.ublast_output_file.txt -threads 4"

**Extract sequence ids contained in column 1 from candidate sequence output files from *ublast***

awk '{if(FILENAME!=prev){close(prev)};print $1 > (FILENAME".ids.txt");prev=FILENAME}' *.txt (or whatever file name is from previous step)

**Extract ublasted candidate sequences from original fastas** <https://bioinf.shenwei.me/seqkit/usage/>**:**

parallel 'cat {} | seqkit grep -f {.}.ids.txt > {.}.output.fa' ::: *fa

**Create a protein database for use with blast:**

makeblastdb -in BacMet2_EXP_database.fasta -dbtype prot -input_type fasta -out db/blast/BacMet2_aa -hash_index

**Create a protein database for use with Diamond blastx** (Buchfink *et al.*, 2015)**:**

diamond makedb --in BacMet2_EXP_database.fasta -d BacMet2

**Diamond blastx all candidate sequences originally derived from USEARCH ublast:**

ls *.fa | parallel --gnu "diamond blastx -q {} -d …/BacMet2.dmnd --max-target-seqs 1 --threads 4 --evalue 0.000001 -o {/.}.diamond.tab"

awk '{if(FILENAME!=prev){close(prev)};print $1 > (FILENAME".diamond_blastx_ids.txt");prev=FILENAME}' *.tab

**Extract final target sequences:**

awk '{if(FILENAME!=prev){close(prev)};print $1 > (FILENAME".ids.txt");prev=FILENAME}' *.tab

**Get counts of each gene per read**

ls *.tab | parallel --gnu "cut -f 2 {} > {/.}.outputfile.txt"

ls *.txt | parallel --gnu "sort {} | uniq -c | sort -n > {/.}.ids_counts"

**Get sequence ids from original BacMet and save to file**

grep '>' …/BacMet2_EXP_database.fasta > file.txt

**Prodigal**

ls *.fasta | parallel prodigal -i {} -o {.}.genes -a proteins{.}.faa -p meta

**Use BBMAP to split large FASTA files for usearch 32-bit version (**<https://jgi.doe.gov/data-and-tools/bbtools/>)

ls *.fasta| parallel --gnu "partition.sh in={} out=out_dir/{/.}part%.fasta ways=5"

**Recombine or concatenate usearch-blastx search results**:

1. touch filename_finish.txt
2. cat file_name_mergedpart* >> filename_finish.txt

**References:**

Buchfink, B., Xie, C., and Huson, D.H. (2015) Fast and sensitive protein alignment using DIAMOND. *Nat Methods* **12**: 59–60.

Edgar, R.C. (2010) Search and clustering orders of magnitude faster than BLAST. *Bioinformatics* **26**: 2460–2461.

Yang, Y., Jiang, X.-T., and Zhang, T. (2014) Evaluation of a Hybrid Approach Using UBLAST and BLASTX for Metagenomic Sequences Annotation of Specific Functional Genes. *PLoS ONE* **9**:.
